# Factors Associated with Information Needs and Information-Seeking Intention Among People with Cancer Experience in Hong Kong

**DOI:** 10.1007/s13187-024-02551-5

**Published:** 2024-12-17

**Authors:** Leanne Chang, Timothy K. F. Fung, Ho Man Leung

**Affiliations:** 1 Department of Communication Studies, Hong Kong Baptist University, KLN, Hong Kong; 2 Department of Interactive Media, Hong Kong Baptist University, KLN, Hong Kong

**Keywords:** Cancer information seeking, Information needs, Survey, Supportive care

## Abstract

Information is crucial for person-centered cancer care. This study investigated sociodemographic, psychological, and communicative factors associated with perceived information needs and the intention to continue seeking information among individuals with cancer experience in Hong Kong. Data were drawn from the INSIGHTS-Hong Kong (International Studies to Investigate Global Health Information Trends) survey, which included 510 respondents with personal cancer experience or as family members and close friends of those diagnosed with cancer. The findings revealed that 62% of participants perceived knowledge deficits and needed more cancer information, yet only 43% intended to seek additional information. Greater cancer worry, extensive effort in previous information searches, and concerns about information quality were significantly associated with heightened information needs. These results highlight key areas for prioritization in educational and supportive care initiatives to address unmet support needs. Additionally, the intention to seek further information was associated with perceived information needs, cancer severity, subjective norms, and concerns about information usefulness. These findings suggest strategies to enhance supportive care services by addressing unmet information needs through expanding access to credible and clear information, enhancing credibility assessment skills, emphasizing cancer risks, and leveraging support networks for individuals affected by cancer. This study lays the groundwork for future research on cancer information engagement in Hong Kong and other settings.

## Introduction

Cancer is the leading cause of death in Hong Kong, accounting for 24% of registered deaths in 2022 [1]. In 2021, Hong Kong had 38,462 newly diagnosed cancer cases, reflecting a notable 12.5% increase from 2020 within its 7.4 million population [2]. The growing threat of cancer and the rising demand for supportive care highlight the need to understand the information needs and engagement of those affected [3–5]. This understanding has implications for enhancing person-centered care and addressing cancer burden at both individual and health system levels [3–5].

Information is critical to cancer care and coping during the cancer journey [6, 7]. Seeking information enables individuals with cancer experience, including those directly diagnosed and their families and friends who gain experience from them, to assess the situation, manage uncertainty, maintain a sense of control and normality, facilitate patient-provider communication, and make informed decisions [3, 6, 8]. When information needs are met, it has positive impacts on treatment planning, adherence, quality care, and well-being [9, 10]. However, unmet information needs are common among individuals with cancer experience [4, 11]. A systematic review found that 31–38% of advanced cancer survivors reported lacking sufficient information, with rates among their informal caregivers reaching up to 38% [4]. Another review of Chinese cancer patients revealed that the prevalence of unmet information needs ranged from 37 to 73% [11]. These reviews examined covariates of unmet information needs, including socio-demographics, cancer characteristics, and health conditions, but found inconsistent or no associations [4, 11], highlighting the need for further research on key influences.

Additional literature indicates that when facing cancer uncertainty, some individuals may seek more information to aid coping, while others adopt a blunting approach to avoid further exposure [3, 8, 12]. This suggests that factors beyond unmet information needs may influence individuals’ motivation to seek more cancer information [3, 12]. Accordingly, this study aims to (1) identify factors associated with perceived needs for more information and (2) examine their relationship with the intention to engage further with cancer information among individuals with cancer experience in Hong Kong.

Drawn from health information-seeking theories, including the Comprehensive Model of Information Seeking [13] and the Planned Risk Information Seeking Model [14], this study examines key theoretical variables that have demonstrated significance in predicting health information needs and information seeking in non-cancer contexts [12, 15, 16]. Specifically, the variables include: (1) sociodemographic characteristics, including gender, age, income, and education; (2) psychological factors, including worry, perceived susceptibility, perceived severity, personal control over health risks, subjective norms, and current knowledge level; and (3) communicative factors, including the ability to access information and perceived usefulness of acquired information [12–16]. While some of these factors have been explored in cancer research, findings remain inconsistent [3, 8, 12, 15, 16, 18]. For instance, younger age, female gender, higher education, and greater income often facilitate cancer information seeking [3, 7, 18], but the effects of cancer worry, perceived cancer risk, perceived norms around staying informed, and perceived control over cancer risk on information needs and seeking behaviors have been mixed [3, 8, 12, 16]. Additionally, studies using the US-based Health Information National Trends Survey (HINTS) highlight frequent communicative barriers in cancer information seeking, including high effort, frustration with the process, difficulty accessing high-quality information, and confusion with the information [18–20]. These negative experiences could hinder continued information engagement among cancer patients and survivors [3, 8]. This study examines these factors to provide an integral understanding of their interplay and offer theory-informed insights for improving the allocation of care resources.

## Methods

### Study Design and Setting

This study used cross-sectional survey data from the International Studies to Investigate Global Health Information Trends (INSIGHTS), an international adaptation of HINTS that has been conducted in Hong Kong and other regions to capture health information trends within a global context [21]. Detailed descriptions of the INSIGHTS project, including its methodology and implementation, have been published elsewhere [21–23].

### Study Population

INSIGHTS-HK targeted Hong Kong residents aged 18 and above [23]. Quota sampling was employed to reflect the gender and age distribution of the 2016 Hong Kong By-Census [24], though the 50 + age group was slightly underrepresented. Our study participants included individuals who had experienced cancer themselves or had family members or close friends with cancer. Of the 1,100 cases in the dataset, 510 respondents (46%) met the criteria and were analyzed. The gender and age distribution of our sample aligned with the census data [24].

### Data Collection

Data were collected online in May–June 2021 through Dynata, a global research company. The online data collection aligned with COVID-19 social distancing measures and followed established industry standards.

### Measures

Survey questions were adapted from the validated 2019 HINTS 5, Cycle 3 and benchmarked against INSIGHTS-China to ensure validity and reliability [22, 25]. Detailed items are included in the Appendix.

#### Sociodemographic Characteristics

Participants’ age, gender, and recoded measure of education (university and above), income (≥ HK$25,000, per Hong Kong median [24]), and health status (good or very good) were obtained.

#### Psychological Variables

Six items assessed worry (“How worried are you about having cancer or recurrence?”); perceived susceptibility (“In your life, how likely do you think you will be diagnosed with cancer or recurrence?”); perceived severity (“How serious do you think it would be if you were diagnosed with cancer or recurrence?”); personal control (“How confident are you about your ability to prevent cancer or recurrence?”); subjective norms (“My family and friends expect me to seek cancer information”); and current knowledge level (“How much do you think you currently know about cancer?”).

#### Communicative Variables

Information accessibility was assessed using two items: effort expended (“It took a lot of effort to get the information you needed”) and frustration during past searches (“You felt frustrated during your search for the information”). Information usefulness was assessed with two other items addressing information quality (“You were concerned about the quality of the information”) and clarity (“The information you found was hard to understand”).

#### Outcome Measures

Perceived information needs were assessed by the extent to which additional knowledge was required (“How much more knowledge would you need to feel confident enough to address the risk of cancer?”). Information-seeking intention was assessed using one item (“I plan to seek cancer information in the near future”).

### Data Recoding

All items, except for sociodemographics, were anchored on 5-point scales and recoded into binary variables (0 = lacking the trait, 1 = having the trait) using the midpoint as the threshold. For instance, perceived information needs were recoded as 0 = low needs and 1 = high needs. Seeking intention was recoded as 0 = no or low intention and 1 = intention to seek information.

### Statistical Analysis

SPSS version 29.0 was used. Cross-tabulations with chi-square tests analyzed sociodemographic, psychological, and communicative factors by information needs. Univariate logistic regression assessed the crude odds ratio of each antecedent with information needs and seeking intention. Significant sociodemographic characteristics and all theoretical factors were included as adjustment variables in the multivariate logistic regression based on univariate significance and theoretical relevance to evaluate their unique effects. Statistical significance was set at p < 0.05.

### Ethical Consideration

The authors’ university Institutional Review Board approved this study protocol.

## Results

### Participant Characteristics

Participants’ ages ranged from 18 to 65 years (*M* = 42.49, *SD* = 11.54). The majority of them were female (56.7%), college graduates (52.0%), had a monthly income below HK$25,000 (approximately US$3,200; 52.0%), and reported good health (61.6%). Most participants gained cancer experience through relatives (47.7%), followed by parents (38.8%), and close friends (22.0%). Overall, 62.0% of participants (*N* = 316) reported high information needs. The only significant sociodemographic difference in perceived information needs was whether participants had relatives with cancer (Table 1).

**Table 1:** Sociodemographic characteristics of participants

Variable	Low Needs	High Needs	Total	*p*-value
	*n* = 194	*n* = 316	*n* = 510	
	*N* (%)	*N* (%)	*N* (%)	
Age				
18–34	47 (24.2)	93 (29.4)	140 (27.5)	.34
35–49	71 (36.6)	116 (36.7)	187 (36.7)	
50 +	76 (39.2)	107 (33.9)	183 (35.9)	
Gender				
Male	85 (43.8)	136 (43.0)	221 (43.3)	.93
Female	109 (56.2)	180 (57.0)	289 (56.7)	
Education				
Middle school or below	8 (4.1)	11 (3.5)	19 (3.7)	.82
High school	53 (27.3)	76 (24.1)	129 (25.3)	
Diploma/Certificate	30 (15.5)	41 (13.0)	71 (13.9)	
Associate degree program	10 (5.2)	16 (5.1)	26 (5.1)	
University	75 (38.7)	141 (44.6)	216 (42.4)	
Graduate School	18 (9.3)	31 (9.8)	49 (9.6)	
Income				
< HK$ 25,000	95 (49.0)	170 (53.8)	265 (52.0)	.19
HK$25,000 – HK$49,999	69 (35.6)	114 (36.1)	183 (35.8)	
> HK$50,000	30 (15.5)	32 (10.1)	62 (12.2)	
Cancer experience				
Myself	14 (7.2)	15 (4.7)	29 (5.7)	.25
Parent	81 (41.8)	117 (37.0)	198 (38.8)	.30
Sibling	14 (7.2)	25 (7.9)	39 (7.6)	.87
Spouse	8 (4.1)	7 (2.2)	15 (2.9)	.28
Child	5 (2.6)	2 (0.6)	7 (1.4)	.11
Other relative	78 (40.2)	162 (51.3)	240 (47.7)	.02
Close friend	45 (23.2)	67 (21.2)	112 (22.0)	.66
Health status				
Very poor	1 (0.5)	3 (0.9)	4 (0.8)	.15
Poor	8 (4.1)	21 (6.6)	29 (5.7)	
Fair	55 (28.4)	108 (34.2)	163 (32.0)	
Good	104 (53.6)	159 (50.3)	263 (51.6)	
Very good	26 (13.4)	25 (7.9)	51 (10.0)	

The cumulative percentage of cancer experience does not total 100% because respondents may have multiple individuals within their circle who have experienced cancer

**Fig. 1:**
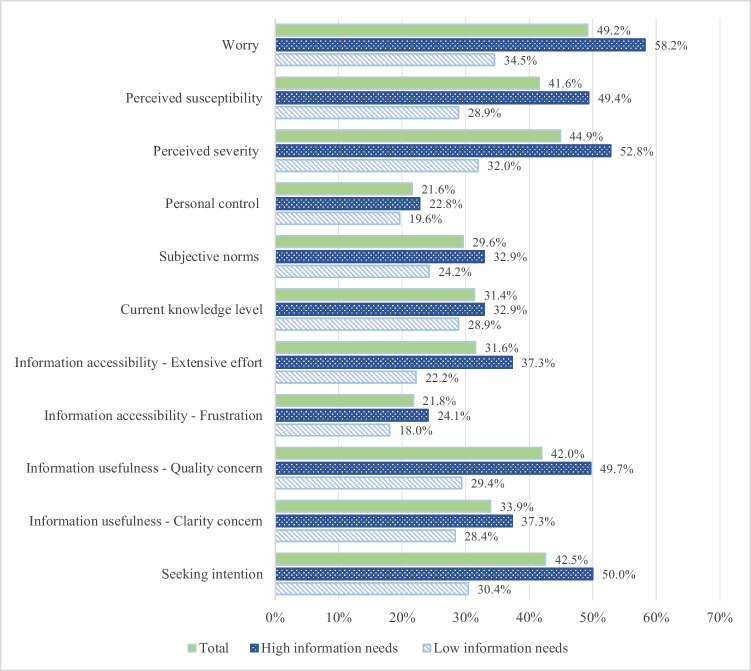
Percentage distribution of psychological and communicative variables by information needs. *Note.* Total participants = 510. High information needs = 316. Low information needs = 194. All variables were binary. The figure shows the percentage of participants reporting the presence of each trait

Figure 1 shows the percentage distribution of psychological and communicative variables by perceived information needs and overall totals. Individuals with greater perceived knowledge deficits and information needs were more likely to worry about cancer development (58.2%, χ^2^ = 26.70, p < 0.001), perceive cancer severity (52.8%, χ^2^ = 21.20, p < 0.001), feel susceptible to cancer (49.4%, χ^2^ = 20.80, p < 0.001), and perceive stronger social norms to stay informed (32.9%, χ^2^ = 4.35, p < 0.05). They reported similarly low personal control over cancer risks (22.8%) and comparable current knowledge levels (32.9%) compared to those with low information needs. These individuals also experienced greater communicative barriers during previous information searches, including concerns about information quality (49.7%, χ^2^ = 20.34, p < 0.001), information clarity (37.3%, χ^2^ = 4.34, p < 0.05), and extensive search efforts (37.3%, χ^2^ = 12.82, p < 0.001). Among participants with high information needs, 50.0% reported plans to seek more information soon (χ^2^ = 18.87, p < 0.001), compared to 30.4% of those with low needs and an overall percentage of 42.5%.

### Factors Associated with Perceived Information Needs

Table 2 shows that poor self-reported health (*OR* = 0.69, p < 0.05), high perceived likelihood (*OR* = 2.40, p < 0.001) and seriousness (*OR* = 2.39, p < 0.001) of developing cancer, strong perceived informational norms (*OR* = 1.53, p < 0.05), and concerns about the clarity of acquired information (*OR* = 1.51, p < 0.05) were univariately associated with the feeling of lacking sufficient knowledge. However, these associations became non-significant after adjusting for covariates. In the multivariate logistic regression model (Nagelkerke’s *R*^2^ = 14.6%, p < 0.001), greater cancer worry (*OR* = 1.72, p < 0.05) was associated with a greater need for knowledge. Additionally, individuals who reported extensive effort in prior searches (*OR* = 1.76, p < 0.05) and concerns about the quality of acquired information (*OR* = 1.97, p < 0.01) were more likely to feel greater information needs.

**Table 2:** Factors associated with perceived information needs and information-seeking intention

Variable	Perceived Information Needs				Information-Seeking Intention			
	Univariate		Multivariate		Univariate		Multivariate	
	*OR* [95% CI]	*p*-value	*OR* [95% CI]	*p*-value	*OR* [95% CI]	*p*-value	*OR* [95% CI]	*p*-value
Sociodemographic characteristics								
Female	1.03 [0.72, 1.48]	.86	NA		0.70 [0.49, 1.00]	.05	0.83 [0.55, 1.26]	.39
Age	0.99 [0.97, 1.00]	.06	NA		1.00 [0.99, 1.02]	.68	NA	
Income	0.82 [0.58, 1.18]	.29	NA		1.17 [0.82, 1.66]	.39	NA	
University	1.30 [0.91, 1.86]	.16	NA		1.08 [0.76, 1.53]	.69	NA	
Health status	0.69 [0.47, 1.00]	.05	0.70 [0.47, 1.05]	.09	1.20 [0.84, 1.73]	.32	NA	
Psychological variables								
Worry	2.62 [1.82, 3.83]	< .001	1.72 [1.11, 2.68]	.02	2.51 [1.75, 3.60]	< .001	1.45 [0.91, 2.32]	.12
Perceived susceptibility	2.40 [1.64, 3.52]	< .001	1.48 [0.93, 2.36]	.10	1.80 [1.26, 2.58]	.001	0.84 [0.52, 1.38]	.50
Perceived severity	2.39 [1.64, 3.47]	< .001	1.34 [0.86, 2.10]	.20	2.47 [1.72, 3.54]	< .001	1.75 [1.09, 2.82]	.02
Personal control	1.21 [0.78, 1.88]	.39	1.26 [0.76, 2.07]	.37	1.69 [1.11, 2.59]	.02	1.25 [0.74, 2.14]	.40
Subjective norms	1.53 [1.03, 2.30]	.04	1.32 [0.84, 2.08]	.24	6.32 [4.14, 9.66]	< .001	4.64 [2.90, 7.41]	< .001
Current knowledge level	1.21 [0.82, 1.78]	.34	0.97 [0.63, 1.50]	.90	1.74 [1.19, 2.54]	.004	1.30 [0.82, 2.06]	.27
Communicative variables								
Information Accessibility—Extensive effort	2.09 [1.39, 3.15]	< .001	1.76 [1.08, 2.89]	.02	2.22 [1.52, 3.24]	< .001	0.82 [0.49, 1.36]	.44
Information Accessibility—Frustration	1.44 [0.92, 2.25]	.11	0.73 [0.42, 1.27]	.27	3.58 [2.30, 5.59]	< .001	1.42 [0.81, 2.49]	.22
Information usefulness—Quality concern	2.37 [1.62, 3.47]	< .001	1.97 [1.25, 3.10]	.003	3.23 [2.23, 4.66]	< .001	1.68 [1.05, 2.67]	.03
Information usefulness—Clarity concern	1.51 [1.02, 2.22]	.04	0.73 [0.44, 1.21]	.22	3.61 [2.46, 5.30]	< .001	1.90 [1.14, 3.16]	.01
Perceived information needs	NA		NA		2.29 [1.57, 3.34]	< .001	1.74 [1.11, 2.73]	.02
Nagelkerke’s *R*^2^			14.6	< .001			32.9	< .001

Adjustment variables in the multivariate models included sociodemographic characteristics based on univariate significance and all psychological and communicative variables based on their theoretical relevance in the health information-seeking literature. Actual age was included as a continuous variable. All other variables were binary

### Factors Associated with Seeking Intention

Univariate analyses showed that male gender (*OR* = 0.70, p < 0.05), cancer worry (*OR* = 2.51, p < 0.001), perceived likelihood of developing cancer (*OR* = 1.80, p < 0.01), perceived control over cancer (*OR* = 1.69, p < 0.05), current knowledge level (*OR* = 1.74, p < 0.01), extensive effort (*OR* = 2.22, p < 0.001), and frustration with prior searches (*OR* = 3.58, p < 0.001) were each associated with higher information-seeking intention. However, these associations became non-significant after adjusting for covariates. In the multivariate logistic regression model (Nagelkerke’s *R*^2^ = 39.2%, p < 0.001), perceived information needs were significantly associated with seeking intention (*OR* = 1.74, p < 0.05). Additionally, individuals perceiving high cancer seriousness (*OR* = 1.75, p < 0.05), strong social expectations to engage with cancer information (*OR* = 4.64, p < 0.001), and concerns about information quality (*OR* = 1.68, p < 0.05) and clarity (*OR* = 1.90, p < 0.05) were more likely to engage in further information seeking.

## Discussion

Previous research underscores the role of information in person-centered care and the prevalence of unmet information needs among cancer patients, survivors, and their associates [5, 6]. This study aimed to deepen the understanding of factors that prompt their needs for additional information and motivate them to seek it. Our descriptive analyses revealed that many individuals with cancer experience in Hong Kong felt cancer threats and worry yet lacked confidence in their ability to control cancer risks. While most of them acknowledged a need for more cancer knowledge, there was a gap between their awareness and intention to seek additional information. Consistent with prior research [18–20], a substantial portion of them reported challenges in obtaining cancer information and concerns about information quality and clarity. Particularly, more barriers were reported regarding information usefulness than accessibility. These findings suggest that cancer support services prioritize improving the quality and clarity of information when addressing individuals’ information needs. Designing educational interventions to enhance patients’ and caregivers’ skills in identifying reliable information from non-clinical sources could also be beneficial.

In our study, individuals’ information needs were predominantly associated with psychological and communicative factors. All psychological variables, except for personal control and current knowledge level, were independently linked to a heightened desire for cancer information, consistent with their significance in extant health information-seeking literature [12, 15]. The non-significance of personal control and current knowledge level likely reflects a general lack of confidence in cancer control and low self-perceived cancer knowledge across individuals, regardless of their desire for more information. The strong significance of worry highlights the critical role of affective responses in shaping information needs and aligns with prior research [12, 16, 17]. With this knowledge, interventions that prioritize addressing emotional concerns over other types of risk judgment may more effectively fulfill individuals’ information needs.

Regarding communicative factors, our findings align with previous research showing that individuals who struggled to find useful information were more likely to perceive information insufficiency [3, 12, 15]. Encouragingly, this perception of knowledge deficits was positively associated with information-seeking intention, suggesting that it facilitates rather than hinders further information seeking [16, 17]. Concerns about information quality were the strongest factor linked to perceived information needs and a key predictor of seeking intention. These findings illustrate the need to guide individuals toward reliable and easy-to-understand information. Strategies could include improving communication with patients and their companies during hospital visits and diversifying provider-patient communication channels to expand access to trustworthy information sources and strengthen informational support.

Among sociodemographic factors, only male gender was independently associated with higher intention to seek additional information, which differs from previous studies [3, 18]. Alternatively, all psychological factors were independently linked to seeking intention, underscoring their importance in interventions promoting continuous knowledge development in cancer care [15, 16]. The unique significance of perceived severity suggests that strengthening individuals’ risk perception of cancer may encourage further information seeking [15]. Additionally, the strong influence of subjective norms, as identified in previous health information-seeking studies [12, 15, 16], suggests interventions targeting both cancer patients and their support networks to enhance sustained information engagement for improved cancer care and coping.

This study has limitations linked to its secondary analysis design. Using a dataset targeting the general public restricts the inclusion of tailored questions and pathology-related variables and limits a more nuanced understanding of information needs and seeking intention among individuals with cancer experience. Nevertheless, this approach enables cross-regional and longitudinal comparisons with other studies utilizing HINTS and INSIGHTS datasets that are valuable to the global literature on cancer informational support. Future research could expand on these findings and examine the impacts of disease-specific factors on cancer information engagement to further enhance support services.

## Conclusion

Information is critical for individuals navigating cancer care and decision-making. This study identified psychological and communicative factors influencing perceived information needs and intention to seek further information. To address information needs, support strategies could involve reducing cancer-related worry, easing information access, and enhancing individuals’ credibility assessment skills. To promote continued information seeking, care services could further improve the outreach of credible and clear information, emphasize cancer risks, and leverage support networks for individuals affected by cancer. Our findings provide a foundation for future research on cancer information engagement in Hong Kong and beyond.

## Data Availability

The data supporting the findings of this study are available from the corresponding author upon request.

## Appendix. Questionnaire

1. Your gender is?
  - Male (1)
  - Female (2)
2. What is your age?
3. In general, would you say your health is... ?
  - Very poor (1)
  - Poor (2)
  - Fair (3)
  - Good (4)
  - Very good (5)
4. What is the highest grade or level of schooling you completed?
  - No formal education taken/kindergarten (1)
  - Primary school (2)
  - Junior secondary (3)
  - Senior secondary (4)
  - Post-secondary Education (Diploma/Certificate) (5)
  - Post-secondary Education (Sub-degree program) (6)
  - Post-secondary Education (degree courses) (7)
  - Post-graduate Education (8)
5. What is your average monthly income?
  - HK$10,000 or less (1)
  - HK$10,000 to $14,999 (2)
  - HK$15,000 to $19,999 (3)
  - HK$20,000 to $24,999 (4)
  - HK$25,000 to $29,999 (5)
  - HK$30,000 to $34,999 (6)
  - HK$35,000 to $39,999 (7)
  - HK$40,000 to $44,999 (8)
  - HK$45,000 to $49,999 (9)
  - HK$50,000 to $54,999 (10)
  - HK$55,000 to 59,999 (11)
  - HK$60,000 or more (12)
6. How worried are you about having cancer (or recurrence)?
  - Not at all worried 1 (1)
  - 2 (2)
  - 3 (3)
  - 4 (4)
  - Extremely worried 5 (5)
7. In your life, how likely do you think you will be diagnosed with cancer (or recurrence)?
  - Very unlikely 1 (1)
  - 2 (2)
  - 3 (3)
  - 4 (4)
  - Very likely 5 (5)
8. How serious do you think it would be if you were diagnosed with cancer (or recurrence)?
  - Very mild 1 (1)
  - 2 (2)
  - 3 (3)
  - 4 (4)
  - Very severe 5 (5)
9. How confident are you about your ability to prevent cancer (or recurrence)?
  - Not confident at all 1 (1)
  - 2 (2)
  - 3 (3)
  - 4 (4)
  - Completely confident 5 (5)
10. My family and friends expect me to seek cancer information.
  - Strongly disagree (1)
  - Disagree (2)
  - Hard to say (3)
  - Agree (4)
  - Strongly agree (5)
11. How much do you think you currently know about cancer?
  - Knowing nothing 1 (1)
  - 2 (2)
  - 3 (3)
  - 4 (4)
  - Knowing very well 5 (5)
12. It took a lot of effort to get the information you needed.
  - Strongly disagree (1)
  - Disagree (2)
  - Hard to say (3)
  - Agree (4)
  - Strongly agree (5)
13. You felt frustrated during your search for the information.
  - Strongly disagree (1)
  - Disagree (2)
  - Hard to say (3)
  - Agree (4)
  - Strongly agree (5)
14. You were concerned about the quality of the information.
  - Strongly disagree (1)
  - Disagree (2)
  - Hard to say (3)
  - Agree (4)
  - Strongly agree (5)
15. The information you found was hard to understand.
  - Strongly disagree (1)
  - Disagree (2)
  - Hard to say (3)
  - Agree (4)
  - Strongly agree (5)
16. How much more knowledge would you need to feel confident enough to address the risk of cancer?
  - Needing nothing more at all 1 (1)
  - 2 (2)
  - 3 (3)
  - 4 (4)
  - Needing a lot more knowledge 5 (5)
17. I plan to seek cancer information in the near future.
  - Strongly disagree (1)
  - Disagree (2)
  - Hard to say (3)
  - Agree (4)
  - Strongly agree (5)
